# A single topical fluralaner application to cats and to dogs controls fleas for 12 weeks in a simulated home environment

**DOI:** 10.1186/s13071-018-2927-0

**Published:** 2018-07-03

**Authors:** Sivaja Ranjan, David Young, Fangshi Sun

**Affiliations:** 1 Merck Animal Health, 2 Giralda Farm, Madison, NJ 07940 USA; 2 David R Young, Young Veterinary Research Services, 213 South Roselawn Avenue, Turlock, CA 95380 USA

**Keywords:** Bravecto®, Cat, *Ctenocephalides felis*, Dog, Efficacy, Flea, Fluralaner, Topical, Simulated home environment

## Abstract

**Background:**

Fluralaner (Bravecto®, Merck Animal Health, Madison, NJ, USA) is a novel isoxazoline that provides up to 12 weeks flea and tick control when administered orally to dogs. Two assessor-blinded studies, one in dogs, the other in cats evaluated the sustained efficacy of a topical fluralaner formulation against fleas in a simulated home environment (SHE).

**Methods:**

Animals were ranked and blocked into groups of two using flea counts completed 24 hours following *Ctenocephalides felis* infestations placed on dogs on Day -64, and on cats on Day -36. Within blocks animals were randomized to a treatment group, 10 animals per group, one group to receive fluralaner spot-on (minimum dose rate for dogs, 25 mg/kg; for cats, 40 mg/kg), the other to be a sham-treated control. Animals were then placed into their SHE, one animal per pen or cage and then infested with 100 *C. felis* at weekly intervals. Dogs were infested from Day -56 through -21 and cats on Days -28 and -21. Fleas were counted and removed from each dog and cat on Day -1. Study animals were then held in clean pens/cages until treatment on Day 0. One day later, after treatment, all animals were returned to their home environment (SHE). Additional 50-flea challenges were placed on each animal on Days 22, 50 and 78. Fleas were counted and replaced on all animals on Day 1 and weekly thereafter for 12 weeks.

**Results:**

Arithmetic mean counts in control-group animals exceeded 10 fleas at all post-treatment assessments except on Days 1, 7 and 14. All control-group animals remained infested at each assessment from Day or 28 through Day 84, thereby validating the challenge methodology. Fluralaner efficacy was 100% on all occasions except for 2 fleas found on 1 dog on Day 1, and 3 fleas on 1 dog on Day 14. One flea was recovered from 1 fluralaner treated cat on Day 1. There were no treatment-related adverse events.

**Conclusion:**

A single application of a topical formulation of fluralaner is well tolerated and highly effective in the prevention of flea infestations of dogs and cats throughout the 12 weeks following treatment.

## Background

By killing fleas and ticks for an extended period following oral administration, the new class of parasiticides, the isoxazolines, have provided a significant advance in the treatment and control of canine ectoparasite infestations. Three compounds of the class, afoxolaner, sarolaner and lotilaner, build on the traditional approach to parasite control by requiring monthly administration [1–3]. One isoxazoline, fluralaner (Bravecto®, Merck Animal Health, Madison, NJ, USA), brings an additional innovation for dogs by providing a sustained 12-week duration of effectiveness from a single oral administration. Fluralaner’s rapid knockdown of fleas and ticks and sustained efficacy has been demonstrated under laboratory and field conditions [4–7]. This extended duration of activity is important as it has been shown that reduced frequency of treatments can be a tool in improving client compliance with veterinary parasite control recommendations [8].

Repeated studies have led to recognition that systemically acting flea control products such as nitenpyram, selamectin, spinosad and the isoxazolines have the potential to provide a more rapid onset and consistency of activity than topically-applied compounds that need to spread across the skin to exert their effects through direct contact with infesting ectoparasites, and which may be affected by a treated animal’s coat and environmental conditions [9–13]. Since the launch of spinosad tablets in 2007 orally administered flea control has been increasingly accepted, and the emergence of the isoxazolines has accelerated this trend. Nonetheless, topical administration of products that are systemically effective against fleas and ticks may still be important for dogs owners who have difficulty in administering oral formulations. This is even more important for cat owners, as cats do not readily accept tablets and may have to be physically restrained for treatment, a task which is often beyond the abilities of many cat owners [14, 15]. Older topically applied compounds which act by contact may also have reduced efficacy because of resistance or other causes of clinical failures [16–19].

There is therefore a need for a long-duration topical product that will provide equivalent effectiveness as the orally administered flea and tick treatments for dogs, but that can be used both in dogs and in cats. A topically administered spot-on formulation of fluralaner (28% w/v) (Bravecto® Topical Solution, Merck Animal Health, Madison, NJ, USA) is available to address this need. Following topical application of this formulation to dogs fluralaner is rapidly absorbed and produces a pharmacokinetic profile that supports its use at the same dose rate (minimum dose rate of 25 mg/kg) as for oral administration [20]. In cats, topical fluralaner is absorbed more rapidly than in dogs and has a shorter half-life indicating that a higher minimum clinical dose rate (40 mg/kg) is indicated [20].

A key step in establishing a label claim for the control of fleas is to demonstrate efficacy under simulated home environment (SHE) conditions. A simulated home environment provides an ongoing flea challenge because adult fleas are applied directly to the animal while maturing juvenile fleas from the home environment provide a continuous challenge throughout the study. The effectiveness of topical formulation of fluralaner in preventing infestations with fleas (*Ctenocephalides felis*) on dogs and cats for 12 weeks (84 days) following a single treatment is therefore investigated in two SHE studies.

## Methods

### Animals and housing

Both studies utilized an assessor-blinded, negative-controlled, randomized complete block design. All general health and treatment site observations, flea infestations and flea counts were performed by masked individuals.

All animals were housed individually (pens for dogs, cages for cats) and maintained indoors in a SHE capable of supporting the flea life-cycle. Each pen or cage contained carpeting as bedding and, to encourage development of non-parasitic life-cycle stages, flea media was applied to the carpet at the time animals were placed in each pen or cage, and thereafter applied weekly for the remainder of the study.

The animals were provided a thermostatically controlled environment with a 12 h light:12 h dark photocycle. Pens and cages were organized so that there was no contact possible between animals, and no possibility of cross-contamination between different treatment groups in either study. Animals were fed an appropriate commercial food, allowed access to water according to study site practice, and provided routine veterinary health care.

For the dog study, 28 healthy intact male and female (non-pregnant and non-lactating) Beagles, older than 6 months and ranging between 8.1–13.1 kg body weight were screened, bathed on Day -70 using a non-medicated shampoo and placed in pens for acclimation. On Day -56, the 24 dogs with the highest flea counts (75–99 live fleas) from an infestation on Day -64 were placed in individual study pens designed to simulate the home environment. Four dogs with the lowest qualifying flea counts were designated as alternates in order to provide replacement dogs should an allocated dog require removal prior to treatment on Day 0.

For the cat study, 26 domestic short- and long-hair male (intact) and female (intact and spayed, non-pregnant and non-lactating) cats, older than 14 weeks and weighing 2.2–5.6 kg, were assigned on Day -42 to individual cages for acclimation. All were bathed on Day -39 using a non-medicated shampoo, infested with fleas on Day -36, combed approximately 24 h later and counts conducted. On Day -28, the 20 cats with the highest Day -35 flea counts were placed in their individual SHE cages.

### Flea challenge and counts

In both studies, fleas used for infestations originated from a colony that was established in 1997 with wild-caught *C. felis* and refreshed periodically with wild fleas from naturally infested animals. For evaluation of susceptibility to experimental infestations and for randomization to treatment groups, *C. felis* infestations were placed on dogs on Day -64 and on cats on Day -36. To establish an environmental flea infestation with a self-perpetuating life-cycle before treatment, each dog was then infested with approximately 100 newly emerged unfed *C. felis* at weekly intervals from Day -56 through -21. Each cat was infested with approximately 100 newly emerged unfed *C. felis* on Days -28 and -21. In addition, to simulate introduction of new fleas into a home environment, each animal, dogs and cats, was infested with 50 newly emerged unfed adult fleas on Days 22, 50 and 78. Thus, once returned to their original cages after treatment on Day 0, it was expected that the developing flea population remaining in the pens or cages, along with the additional 50-flea infestations on Days 22, 50 and 78 would provide adequate flea challenge, similar to what would occur naturally in a home environment.

Flea counts were completed using a flea comb (26 teeth per inch) in overlapping strokes from the front (head, ears, neck, etc.) to the back of the animal, including the tail, lateral sides, legs, chest and ventral sides. Each animal was combed until no fleas were recovered in a period of 5 min. All fleas recovered were removed from the comb by hand and classified as live or dead, and the total live flea count recorded.

Once enrolled in the study, flea counts were completed on Day -1 (the day before treatment), Day 1 (the day after treatment), and thereafter weekly until completion of the study on Day 84. Live fleas recovered on Day -1 were not placed back on the animals which were held in clean cages overnight. All live fleas recovered at each subsequent combing were held in a suitable container and returned to the animal at the conclusion of the count.

### Randomization and treatment

The 24 dogs with the highest Day -63 live flea counts were placed into study pens. Of these, the 20 dogs with the highest Day -63 counts were blocked into groups of two, and each dog within a block was randomized to one of the two treatment groups, either a fluralaner treated group or a sham-treated control group, each group consisting of 10 dogs. The remaining four dogs were designated as alternates for use if replacement of one of the 20 dogs with the highest flea counts was needed. On the Day -1 (pre-treatment) assessment of infestations, a total of 6 dogs enrolled in the study, 2 dogs from control group and 4 from the group to receive fluralaner had fewer than 5 live fleas, while 3 alternate dogs had more than 5 fleas each. One of the low-flea-count dogs in the control group and two in the fluralaner group were randomly selected to be replaced by randomly selected dogs from the alternate pool which had more than 5 fleas. To maintain adequate numbers of dogs for the efficacy assessment, two fluralaner-group dogs and one control group dog with fewer than 5 fleas on Day -1 were therefore retained in the study.

The 20 cats with the highest Day -35 counts were blocked into groups of two and randomized to either the fluralaner group or the sham-treated control group (10 cats per group). One cat in the control group was removed from the study on Day -3 for ill-health and was not replaced, leaving 9 cats in the control group.

Fluralaner was applied topically using a calibrated syringe between the shoulder blades for dogs and at the base of the skull for cats. The minimum clinical dose rates were 25 mg/kg for dogs and 40 mg/kg for cats. Animals in the negative control groups were sham-treated with an empty syringe to simulate treatment with the active product, in order to maintain similar handling of animals in both groups and to provide a reference time for post-treatment activities. Each animal was kept on the treatment table for approximately 5 min following administration and monitored for any potential abnormal occurrence before being returned to its simulated environment.

In both studies, the dorsal midline, the product application site (including the base of skull for cats) of all animals was examined before treatment (Day -2), at approximately 24 and 48 h after treatment, and thereafter at weekly intervals until the completion of the study. The health of all study animals was checked at 1, 3 and 6 h post-treatment and at least once daily through the end of the study. All animals were closely monitored for any adverse event, defined as any observation that was unfavorable and unintended that occurred after treatment, whether or not it was considered to be treatment related.

### Statistical assessments

The individual dog or cat was the experimental unit. Evaluation of effectiveness was considered valid for both studies if at least 1 live flea was counted on at least 6 control animals at each evaluation. Adequacy of infestations was viewed as demonstration of a self-replicating flea cycle in each pen/cage. Data at each time point were analyzed separately. Flea count data were transformed prior to analysis using the Y = log_e_(x+1) transformation. Log-transformed data were analyzed by a mixed linear model including treatment as the fixed effect and block as the random effect. Least squares means were used for treatment comparisons and were back-transformed to obtain the estimates of geometric mean flea counts. A Kenward-Rogers adjustment was used to determine the denominator degree of freedom for hypothesis.

A two-tailed t-test was used for the comparison between treatment groups. Statistical significance was declared when *P* ≤ 0.05. The primary software used was SAS version 9.3 (SAS Institute Inc., Cary, NC, USA, Release 9.3).

Efficacy was calculated using arithmetic and geometric means with Abbott’s formula:

$$ \mathrm{Efficacy}\left(\%\right)=100\times \left({\mathrm{M}}_{\mathrm{C}}\hbox{-} {\mathrm{M}}_{\mathrm{T}}\right)/{\mathrm{M}}_{\mathrm{C}} $$

where M_C_ is the mean number of total adult live fleas on untreated dogs/cats and M_T_ is the mean number of total adult live fleas on treated dogs/cats.

## Results

The dogs and cats included in the studies demonstrated susceptibility for flea infestations based on flea counts at enrollment. The flea count of all enrolled dogs ranged between 75–99 live fleas on Day -63. The flea counts of all enrolled cats ranged between 63–92 on Day -35. Topical fluralaner was applied in a single spot in volumes ranging between 0.7–1.2 ml per dog; and between 0.3–0.8 ml per cat, representing the minimum clinical dose of 25 mg/kg for dogs and 40 mg/kg for cats. Product loss or run-off was not observed at the treatment site from any dogs or cats. There were no abnormal treatment site observations nor any product-related adverse events in any animal in either study.

Pre-treatment live flea counts (Day -1) from enrolled dogs ranged between 0–98. Arithmetic mean live flea counts on control dogs ranged from 4.0 (Day 14) to 58.0 (Day 84). On all post-treatment count days, there were at least 8 control dogs with one or more live fleas, and all control dogs remained infested at each assessment from Day 28 through Day 84, thereby establishing the adequacy of infestation (Table 1). In the fluralaner-treated group, 2 fleas were found on 1 dog the day after treatment, and 3 fleas were found on another dog 14 days after treatment. No fleas were found on any fluralaner treated dog at any other post-treatment assessment. Therefore in dogs, topical fluralaner flea control efficacy in this SHE model was 100% on all count days except for 96.0% on Day 1 and 94.1% on Day 14 (Table 1). Flea counts on fluralaner treated dogs were significantly lower than control group counts at the first post-treatment assessment, (t-test: *t*_(9.0)_ = -4.198, *P* = 0.002) and remained significantly lower on all post-treatment days through the final assessment on Day 84 (t-test: *t*_(9.0)_ = -28.25, *P* < 0.0001).

**Table 1:** Flea-control efficacy fluralaner-treated dogs compared to sham-treated controls in a simulated home environment

Days pre/post-treatment	Mean flea count (arithmetic/geometric)		% Efficacy (arithmetic/geometric)	*t*_( *df*)_	*P*
	Control group^a^	Fluralaner group^a^			
-1	41.6/24.2 (10/10)	19.4/10.4 (8/10)	na		
1	4.4/2.9 (8/10)	*0.2/0.1 (1/10)	95.5/96.0	*t*_(9.0)_ = -4.198	0.002
7	5.2/4.0 (10/10)	**0.0/0.0 (0/10)	100/100	*t*_(9.0)_ = -7.425	< 0.0001
14	4.0/2.5 (9/10)	**0.3/0.1 (1/10)	92.5/94.1	*t*_(18.0)_ = -3.740	0.001
21	26.7/12.6 (8/10)	**0.0/0.0 (0/10)	100/100	*t*_(9.0)_ = -4.947	0.001
28	27.8/19.9 (10/10)	**0.0/0.0 (0/10)	100/100	*t*_(9.0)_ = -9.925	< 0.0001
35	31.8/30.3 (10/10)	**0.0/0.0 (0/10)	100/100	*t*_(9.0)_ = -33.26	< 0.0001
42	17.7/15.0 (10/10)	**0.0/0.0 (0/10)	100/100	*t*_(9.0)_ = -15.80	< 0.0001
49	34.8/32.8 (10/10)	**0.0/0.0 (0/10)	100/100	*t*_(9.0)_ = -30.95	< 0.0001
56	47.5/40.2 (10/10)	**0.0/0.0 (0/10)	100/100	*t*_(9.0)_ = -18.43	< 0.0001
63	21.9/15.4 (10/10)	**0.0/0.0 (0/10)	100/100	*t*_(9.0)_ = -10.72	< 0.0001
70	12.3/8.9 (10/10)	**0.0/0.0 (0/10)	100/100	*t*_(9.0)_ = -10.49	< 0.0001
77	40.4/37.7 (10/10)	**0.0/0.0 (0/10)	100/100	*t*_(9.0)_ = -32.10	< 0.0001
84	58.0/52.8 (10/10)	**0.0/0.0 (0/10)	100/100	*t*_(9.0)_ = -28.25	< 0.0001

*Abbreviations*: *df* degrees of freedom, *na* not applicable (pre-treatment)

^a^Number infested/total number in group

Significantly different from control group: *P = 0.002, **P ≤ 0.001

In the cat study, the removal of a control cat for health reasons prior to Day 0 left ten fluralaner-treated cats and nine control cats for data analysis and efficacy determination. Pre-treatment live flea counts (Day -1) of cats ranged between 0–88. In the control group there were at least six (6–9) control cats with ≥ 2 live fleas except for Days 7 and 14, and all 9 control cats were infested from Days 28 through the final assessment on Day 84 (Table 2). Flea control efficacy of fluralaner treatment was 100% on all days other than 96.1% on Day 1 (Table 2). Except for Days 7 and 14 when too few control group cats had an adequate infestation to allow statistical comparisons, flea counts in the treated group were significantly lower than in the control group on the day after treatment (t-test: *t*_(9.2)_ = -3.447, *P* = 0.007) and remained significantly lower through the final assessment on Day 84 (t-test: *t*_(8.7)_ = -24.18, *P* < 0.0001).

**Table 2:** Flea-control efficacy fluralaner-treated cats compared to sham-treated controls in a simulated home environment

Days pre/post-treatment	Mean flea count (arithmetic/geometric)		% Efficacy (arithmetic/geometric)	*t*_( *df*)_	*P*
	Control group^a^	Fluralaner group^a^			
-1	18.4/15.9 (9/9)	25.9/10.6 (9/10)	na	*–*	–
1	2.9/1.9 (6/9)	*0.1/0.1 (1/10)	96.5/96.1	*t*_(9.2)_ = -3.447	0.007
7	0.7/0.4 (3/9)	0.0/0.0 (0/10)	na	–	–
14	0.8/0.4 (3/9)	0.0/0.0 (0/10)	na	–	–
21	17.1/12.1 (8/9)	**0.0/0.0 (0/10)	100/100	*t*_(8.7)_ = -7.603	< 0.0001
28	36.6/33.3 (9/9)	**0.0/0.0 (0/10)	100/100	*t*_(8.7)_ = -24.84	< 0.0001
35	19.2/17.1 (9/9)	**0.0/0.0 (0/10)	100/100	*t*_(9.0)_ = -17.77	< 0.0001
42	12.2/9.9 (9/9)	**0.0/0.0 (0/10)	100/100	*t*_(9.0)_ = -11.31	< 0.0001
49	11.0/10.0 (9/9)	**0.0/0.0 (0/10)	100/100	*t*_(8.7)_ = -16.51	< 0.0001
56	23.1/21.5 (9/9)	**0.0/0.0 (0/10)	100/100	*t*_(8.7)_ = -24.77	< 0.0001
63	16.8/15.4 (9/9))	**0.0/0.0 (0/10)	100/100	*t*_(8.7)_ = -20.99	< 0.0001
70	16.4/12.9 (9/9)	**0.0/0.0 (0/10)	100/100	*t*_(8.7)_ = -11.24	< 0.0001
77	17.7/14.9 (9/9)	**0.0/0.0 (0/10)	100/100	*t*_(8.7)_ = -14.94	< 0.0001
84	37.0/32.7 (9/9)	**0.0/0.0 (0/10)	100/100	*t*_(8.7)_ = -24.18	< 0.0001

*Abbreviations*: *df* degrees of freedom, *na* not applicable pre-treatment or < 6 control cats were infested and efficacy not calculated

^a^Number infested/total number in group

Significantly different from control group: *P = 0.007, **P ≤ 0.001

## Discussion

A single application of a topical formulation of fluralaner at the minimum clinical dose was highly effective for controlling flea infestations on dogs and cats in a SHE for 12 weeks following treatment. Under these SHE conditions the rapid onset of the protective effect of topical fluralaner against flea infestations on both dogs and cats was confirmed, and the efficacy found in these studies confirms reports of fluralaner efficacy in dogs and cats, both under SHE conditions and in naturally infested client-owned dogs and cats [5, 21]. The results can be attributed to the systemic fluralaner pharmacokinetic profile reported to follow topical or oral administration [20, 22].

The results achieved by our single treatment of dogs with fluralaner are consistent with findings of the flea control reported from other SHE studies. In a shorter study (60 days), dogs were free of fleas following two consecutive monthly oral treatments with sarolaner, and in another study over 90 days in which dogs received three consecutive monthly applications of selamectin [23, 24]. In cats, our results also align with two other reports of SHE studies, one describing three consecutive monthly applications of selamectin, and another in which cats received six consecutive monthly treatments with imidacloprid or fipronil [24, 25]. All these consecutive monthly treatments produced a high level of flea control under SHE conditions, but under real-world conditions the reliability of owner adherence to repeated monthly treatments is questionable [26]. The cat flea is a prolific egg layer and, thus, is well adapted for infesting homes and dwellings so that a missed or delayed treatment has the potential to result in a resurgence in environmental contamination with developing flea life-cycle stages [4]. The challenge methodology employed in SHE model used in the studies reported here is very intensive because it includes adult fleas maturing from life stages in the environment as well as repeated additional challenge with live fleas to mimic infestation acquired outside the home environment, similar to natural challenge. Fluralaner efficacy was 100% at most assessment time points for dogs and cats, with only a very low number of fleas found sporadically on treated animals, at the early assessment times of the study.

The time period before treatment to establish flea infestations in the simulated home environment was longer for dogs (56 days) than for cats (28 days). In both studies, the steady increase in control group mean flea counts during the study period indicated that flea populations were established and maintained in this simulated contaminated natural environment and provided sustained adequate challenge.

No adverse events were seen in any of the treated dogs or cats in this study. Additionally, there were no treatment site problems with no evidence of either run-off (treatment spread through the hair) nor drip-off (treatment solution leaving the animal) from the treated animals.

## Conclusions

A single topical administration of fluralaner at the minimum clinical dose is highly effective for protecting both dogs and cats against an intensive flea challenge through 12 weeks post-treatment.

## Abbreviations

BW: body weight
M_C_: mean number of total adult live fleas on untreated dogs/cats
M_T_: mean number of total adult live fleas on treated dogs/cats
SHE: simulated home environment
